# Calcineurin Inhibitor-Induced Type IV Renal Tubular Acidosis in Post-Bone Marrow Transplant: Review of Pathophysiology and Principles of Management

**DOI:** 10.7759/cureus.40215

**Published:** 2023-06-10

**Authors:** Anwar AL-Omairi, Mohammed Abdulnasser Obaid, Indira Agrawal, Amr Abdalla, Abdul Hakim Al Rawas

**Affiliations:** 1 Child Health, Sultan Qaboos University Hospital, Muscat, OMN

**Keywords:** drug induced hyperkalemia, nephrotoxic drugs, renal tubular acidosis type 4, cyclosporine, post bone marrow transplant complications

## Abstract

Calcineurin inhibitors (CNI) are the mainstay of immunosuppressant medications in both bone marrow transplants and solid organ transplants. Nephrotoxicity is a well-known adverse effect of this group. Type IV renal tubular acidosis is a potentially under-recognized complication. Here we report a case of Omenn syndrome in a patient who underwent a bone marrow transplant and developed type IV renal tubular acidosis while on treatment with cyclosporine.

## Introduction

Calcineurin inhibitors (CNI) are the mainstay of immunosuppressant therapy in both bone marrow transplants and solid organ transplants. Their introduction in the field of transplants has led to major advancements in graft survival [1,2]. They suppress the immune system by decreasing the production of interleukin-2 (IL-2), which is a pivotal step in T cell proliferation [3]. Type IV renal tubular acidosis (RTA) is a rare complication of CNI therapy that is potentially under-recognized. It is associated with hyperkalemic normal anion gap metabolic acidosis. Failure to recognize the condition may result in a catastrophic outcome arising from hyperkalemia. We report a case of Omenn syndrome in a patient who underwent a bone marrow transplant and developed type IV renal tubular acidosis while on treatment with cyclosporine. The aim of this report is to review the pathophysiology and mechanism of action of CNIs and to share the treatment strategies necessary to mitigate the life-threatening risks associated with hyperkalemia.

## Case presentation

We present a male child diagnosed with severe combined immunodeficiency at the age of six months. He presented with failure to thrive, extensive eczema, and recurrent pneumonia with a history of pneumocystis jiroveci pneumonia. His genetic testing revealed a mutation in the DCLRE1C gene consistent with the diagnosis of Omenn syndrome. He underwent a bone marrow transplant (BMT), the donor being his father, who had haploidentical T cell receptor alpha, beta, and CD19 depletion. The source of stem cells was the peripheral blood, with a dose of 10 × 106/kg. The conditioning was made by thiotepa, treosulfan, fludarabine, and anti-thymocyte globulin. He achieved complete chimerism by day 20. However, seven months post-BMT, he developed normal anion gap metabolic acidosis associated with hyperkalemia and a mild elevation in serum creatinine. He didn't have diarrhea, and he was not on IV fluids. On examination, his BP was 98/66 mmHg, respiratory rate (RR) was 24/min, T was 36.5, and oxygen saturation was 100% in room air. He appeared well and active. He had no signs of dehydration or respiratory distress. His chest was clear, and his cardiovascular, neurological, and abdominal examinations were normal. His medication list included Cyclosporin, Penicillin, Folic Acid, Ursodeoxycholic, Acyclovir, Magnesium Oxide, a prophylactic dose of Cotrimoxazole, and monthly infusions of intravenous immunoglobulin.

Lab work is shown in Tables 1, 2. Abdomen ultrasound showed both kidneys had normal parenchymal echogenicity, with no evidence of renal stones or hydronephrosis. Since he has no other causes of normal anion gap metabolic acidosis, such as diarrhea or being on IV normal saline, transtubular potassium gradient (TTKG) was suggestive of impaired aldosterone action on the distal tubule. He was diagnosed with type IV renal tubular acidosis (RTA) secondary to cyclosporine. He was managed by increasing fluid intake to 100 ml/kg/day, sodium bicarbonate orally at 1 mmol/kg twice a day (BID), and a low potassium diet. Follow-up lab results within one week showed normal serum potassium with no metabolic acidosis (Table 1). 

**Table 1:** Serum chemistry HCO3: Bicarbonate

	Before treatment	After treatment	Reference range
Sodium	138	136	(135 to 145) mmol/L
Potassium	6.0	4.3	(3.5 to 5.1) mmol/L
Chloride	110	101	(98 to 107) mmol/L
Creatinine	37	24	(15 to 31) umol/L
Urea	3.9	5.6	(2.8 to 8.1) mmol/L
Osmolality	286	-	(275 to 295) mOsmol/kg
PH	7.21	7.39	(7.35 to 7.45)
CO2	38	33.5	(35 to 48) mmHg
HCO3	11	24	(21.8 to 26.9) mmol/L
Cyclosporine pre-dose level	116	127	(80 to 300) nmol/L

**Table 2:** Urine Electrolytes¥ Transtubular potassium gradient* Normal values of urine sodium and potassium are variable according to diet intake, urine osmolality range: 40 to 1400 mOsmol/kg¥

Sodium	29 mmol/L
Potassium	15 mmol/L
Urine osmolality	273 mOsmol/kg
Calculated *TTKG (< 7 in presence of hyperkalemia suggestive of hypoaldosteronism)	3
PH (4-8)	6.5
Glucose	Nil
Protein	Nil

## Discussion

Calcineurin inhibitors are immunosuppressant drugs. Their main mechanism of action is the inhibition of the phosphatase activity of calcineurin enzymes in T cells. This interferes with the translocation of the nuclear factor of activated T cells (NFAT). NFAT regulates gene expression in T lymphocytes and eventually leads to increased production of IL-2. IL-2 is the main interleukin that activates T cells and stimulates their proliferation. Thus, calcineurin inhibitors exert their immunosuppressant effect through the inhibition of T-cell activation and proliferation [4,5]. Calcineurin inhibitors have other physiological effects, for example, vasoconstriction, which occurs through several mechanisms such as activation of the renin-aldosterone-angiotensin system, reduction of nitric oxide and prostaglandin E2, and increased endothelin production [6,7]. This vasoconstriction effect manifests as an elevation in blood pressure and acute kidney injury.

Another potentially life-threatening effect of cyclosporine is type IV RTA, characterized by normal anion gap hyperkalemic metabolic acidosis. Animal studies have shown that treatment with CNI leads to a decrease in the chloride bicarbonate anion exchanger CL-/HCO3- at the basolateral membrane and the (ATP6V0A4) subunit of the vacuolar H+-ATPase in the apical membrane [8]. A defect in these units is associated with distal renal tubular acidosis and impaired H+ secretion. In addition, there is evidence that treatment with CNI is associated with a state of hypoaldosteronism in the presence of a normal aldosterone level. Several in vitro studies have demonstrated a negative effect of tacrolimus, which is a new CNI, on the number of mineralocorticoid receptors, resulting in type IV RTA [9,10].

Management of type IV renal tubular acidosis consists of decreasing potassium intake, correcting metabolic acidosis, intravascular volume expansion, and increasing sodium intake.

**Figure 1:**
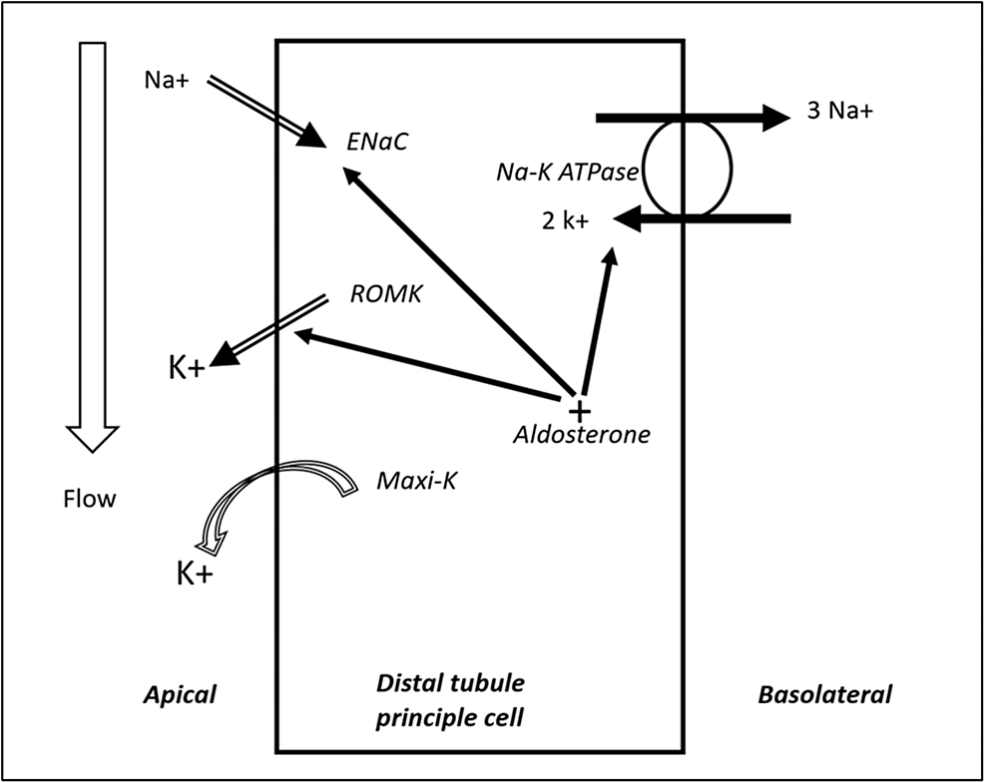
Mechanism of potassium secretion in distal renal tubule Author's own creation

Since a balanced, healthy diet usually contains more than the daily required intake, decreasing oral potassium intake will likely mitigate the risk of overt hyperkalemia. After meals, the body's first defense mechanism against hyperkalemia is to shift potassium into the intracellular compartment. This process is largely impaired by the presence of metabolic acidosis. Therefore, to enhance the shifting of potassium to the intracellular compartment, it is important to correct the metabolic acidosis first. Then it is the role of the kidneys to gradually excrete the excess potassium in the urine. Consequently, intravascular volume expansion would facilitate renal potassium secretion by increasing GFR and flow to the distal renal tubule. This further enhances potassium secretion by two mechanisms (Figure 1). First, washing potassium maintains gradients between the intracellular and luminal compartments, a therapy that enhances and facilitates potassium secretion. Second, flow activates the Maxi-K flow-dependent channels, which secrete potassium. In addition to correction of metabolic acidosis and intravascular volume expansion, it is important to increase sodium intake to deliver more sodium to the distal renal tubule. The presence of sodium in distal tubules facilitates the movement of sodium into principal cells and the movement of potassium out of the cells [11].

**Table 3:** Management of hyperkalemic normal anion gap metabolic acidosis GFR: glomerular filtration rate, ENaC: Epithelial sodium channels

Management	Mode of action
Expand intravascular volume	Increased GFR
Increase sodium intake	Movement of sodium through ENaC creates negative charge in the distal renal tubule and facilitates secretion of potassium
Correction of metabolic acidosis	Facilitates intracellular shift of post-prandial potassium load
Decrease dietary potassium	Decrease the risk of sudden increase in serum potassium

## Conclusions

Calcineurin inhibitors can cause type IV renal tubular acidosis. The hallmark of type IV RTA is the presence of hyperkalemic normal anion gap metabolic acidosis. Early recognition and prompt intervention may be lifesaving. The main principle of management is to decrease potassium intake by shifting it to the intracellular compartment and facilitating renal excretion of potassium. This can be achieved by a low potassium diet, expanding intravascular volume by giving plenty of fluids and sodium bicarbonate supplements to correct acidosis, shifting potassium to the intracellular compartment, and facilitating the excretion of potassium in the distal renal tubule.
